# Analysis of in vitro fertilization/intracytoplasmic sperm injection outcomes in infertile women with a history of thyroid cancer: a retrospective study

**DOI:** 10.1186/s12958-021-00763-8

**Published:** 2021-06-04

**Authors:** Ning Huang, Lin Zeng, Jie Yan, Hongbin Chi, Jie Qiao

**Affiliations:** 1 Center for Reproductive Medicine, Department of Obstetrics and Gynecology, Peking University Third Hospital, 49 North Garden Rd, Beijing, 100191 China; 2 Clinical Epidemiology Research Center, Peking University Third Hospital, Beijing, 100191 China

**Keywords:** Thyroid cancer, Female infertility, In vitro fertilization/intracytoplasmic sperm injection outcome

## Abstract

**Background:**

Recent studies have revealed that women with infertility have a higher risk of thyroid cancer (TC) than fertile women. However, studies on whether a history of thyroid cancer affects clinical outcomes in women who conceive using in vitro fertilization/intracytoplasmic sperm injection (IVF/ICSI) are scarce. We investigate whether a history of thyroid cancer (TC) affects the in vitro fertilization/intracytoplasmic sperm injection (IVF/ICSI) outcomes and increases the risk of adverse obstetric outcomes in women with infertility.

**Methods:**

This retrospective study enrolled 384 women with infertility who underwent their first IVF/ICSI treatment at the Peking University Third Hospital between 2010 and 2019. Participants were divided into the TC (64 women with TC history) and control (320 women matched from 85,272 women without thyroid diseases) groups. Controls were individually matched to the TC group according to age, body mass index, concomitant infertility factors, first IVF/ICSI dates, and controlled ovarian stimulation and embryo transfer procedure protocols. IVF/ICSI outcomes, including the numbers of retrieved oocytes and high-grade embryos, clinical pregnancy, miscarriage, preterm delivery, and live birth rates, and adverse obstetric outcome risk were assessed.

**Results:**

The TC group had significantly higher thyroid hormone and lower thyroid-stimulating hormone (TSH) levels than the control group. Despite similar gonadotropin treatment dosage, the TC group had a significantly lower numbers of retrieved oocytes and high-grade embryos than the control group. The occurrence rates of clinical pregnancy, miscarriage, preterm delivery, live births, and adverse obstetric outcomes, including multiple gestation, preterm delivery, gestational diabetes mellitus, gestational hypertension, low birth weight, and large-for-gestational-age infants, were not significantly different between the two groups.

**Conclusions:**

TC history did not affect the pregnancy outcomes or increase the risk of adverse obstetric outcomes after the first IVF/ICSI, but it may decrease the number of retrieved oocytes and high-grade embryos.

## Background

The incidence of thyroid cancer (TC) is gradually rising globally and is approximately three-fold higher in women than in men [1]. Current studies suggest that the risk of TC is higher in infertile women than in fertile women. In a large-scale, prospective cohort study, Ros et al. recruited 508 women with differentiated TC and observed a significantly higher risk of TC in women with a history of infertility [2]. Recently, another large, retrospective cohort study conducted in Taiwan included 13,356 infertile women and 53,424 fertile women aged between 20 and 50 years and found that the incidence of TC was nearly two-fold greater in infertile women than in fertile women [3].

Pregnancy is an important consideration when treating TC in women of childbearing age. A careful balance of the risks and benefits is needed between the treatment of cancer and maintenance of pregnancy. Hypothyroidism induced by thyroidectomy and hyperthyroidism triggered by thyroid hormone replacement after thyroidectomy may affect thyroid hormone levels and increase the risk of adverse pregnancy outcomes [4]. Reproductive counseling and assisted reproductive technology are important components of the care provided to patients with TC complicated by infertility. Most of the existing studies focus on the association between a history of TC and adverse pregnancy outcomes in women of childbearing age; however, studies evaluating the impact of a history of TC on in vitro fertilization/intracytoplasmic sperm injection (IVF/ICSI) outcomes in women with infertility are lacking.

By focusing on IVF/ICSI, this study analyzed the effect of TC during the earlier gestational stages from oocyte fertilization to embryo implantation and throughout pregnancy. The aim of this retrospective study is to investigate whether a history of TC affects the IVF/ICSI outcomes and increases the risk of adverse pregnancy outcomes.

## Methods

### Study population

**Fig. 1:**
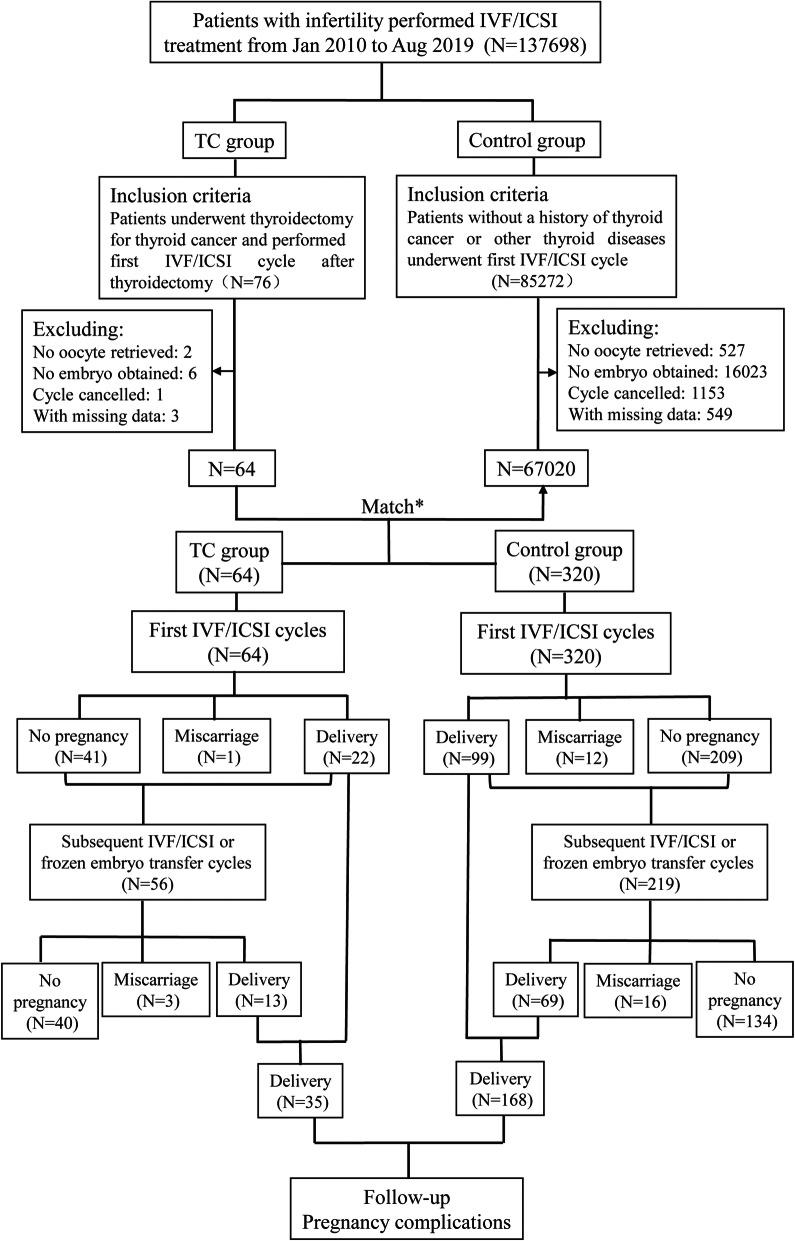
Flow chart of study cohort selection and follow-up procedures. Abbreviation: IVF/ICSI, in vitro fertilization/intracytoplasmic sperm injection

A total of 137,698 patients with infertility referred to the Reproductive Center of Peking University Third Hospital for in vitro fertilization and embryo transfer (IVF-ET) between January 2010 and August 2019 were screened for eligibility (Fig. 1). This study identified 76 patients with infertility and a history of thyroidectomy as the treatment for TC who underwent their first IVF/ICSI treatment after surgery. All patients had been diagnosed with papillary thyroid carcinoma. Altogether, 64 of these patients who had embryo transfer were included in the TC group. A total of 67,020 patients who had embryo transfer were assigned to the control group from 85,272 patients without a history of TC or other thyroid diseases who underwent their first IVF/ICSI treatment cycle. Women in the control group were individually matched to a single case at a ratio of 5:1 for age (±1), body mass index (BMI) (±1), concomitant infertility factors (polycystic ovarian syndrome, endometriosis, tubal factor, or male factor), date of first IVF/ICSI, protocols for controlled ovarian stimulation (COS; ultralong-term, long-term, antagonist, short-term, or mini-stimulation protocol), and ET procedures (fresh embryo or frozen ETs). A total of 320 patients were screened in the control group for data analysis. After first embryo transfer, 28 patients in the TC group and 100 patients in the control group entered into subsequent IVF/ICSI or frozen embryo transfer cycles. All couples were tested for sexually transmitted diseases before IVF/ICSI treatment because of negative influence of sexually transmitted diseases on IVF/ICSI outcomes [5, 6]. All patients, including the TC and control groups, were interviewed by phone on the expected date of delivery to follow-up on details regarding the pregnancy outcomes and complications. The study was approved by Peking University Third Hospital Medical Science Research Ethics Committee.

### IVF/ICSI treatment

All patients underwent a standardized ovarian stimulation regimen, oocyte retrieval, and fertilization, followed by a planned transfer of fresh or frozen embryos. Patients treated with the ultra long-term and long-term protocols received a long-acting gonadotropin-releasing hormone (GnRH) agonist on the 1st and 2nd day of their menstrual cycle and 1 week before the expected day of menstruation, respectively. After downregulation was complete, recombinant gonadotropins were administered for ovarian stimulation. Patients treated with the short-term protocol were administered a short-acting GnRH agonist and recombinant gonadotropins simultaneously on the 2nd through to the 3rd day of the menstrual cycle for ovarian stimulation. In patients treated with the antagonist protocol, recombinant gonadotropins were initiated on the 2nd day of the menstrual cycle, and treatment with a GnRH antagonist was initiated when at least one follicle reached 12 mm in diameter and was continued until the day human chorionic gonadotropin (HCG) was administered. In patients treated with the mini-stimulation protocol, clomiphene was administered orally, and then low-dose gonadotropins were administered for ovarian stimulation. A GnRH antagonist was also administered until the day of HCG administration.

Recombinant HCG at a dose of 5000–10,000 IU was administered to trigger oocyte maturation when ≥2 follicles measuring ≥18 mm, and oocyte retrieval was performed 34–36 h later. The oocytes were inseminated approximately 4–6 h after oocyte retrieval by either IVF or ICSI. Assessment of fertilization is carried out about 16–18 h after insemination and was evaluated by the presence of two pronuclei. Embryo quality were assessed 68–72 h after insemination based on the number, size, symmetry of blastomeres and the percentage of fragmentation according to the criteria established by the Istanbal Consensus Workshop on Embryo Assessment [7]. Good-quality embryos all developed from two pronuclei zygotes and met the following criteria: (1) had > 5 blastomeres; (2) the size difference was < 20%; and (3) fragmentation was < 50%. At least one, and up to three high-quality day-3 embryos or blastocysts were transferred to the patients 3 or 5 days after the oocyte retrieval. Two patients received preimplantation genetic testing (PGT) because of abnormal karyotyping. Some patients with a high risk of ovarian hyperstimulation syndrome underwent frozen ETs.

### Laboratory testing

The blood samples for thyroid hormone testing were taken within 6 months prior to the initiation of COS. Serum thyroid-stimulating hormone (TSH), free thyroxine (FT4), thyroid peroxidase antibody (TPOAb), and thyroglobulin antibody (TGAb) were measured by a fully automatic chemiluminescence immunoassay analyzer (ADVIA Centaur XP, Siemens Healthcare Diagnostics). The reference values for TSH and FT4 were 0.55–4.78 uIU/ml and 0.89–1.80 ng/dl, respectively. A value < 60 IU/ml was defined as negative for TPOAb and TGAb. Basal hormones, including follicle-stimulating hormone (FSH), luteinizing hormone (LH), and estradiol were measured on the 2nd through to the 4th day of the menstrual cycle.

### Study outcomes

Pregnancy outcomes in patients who underwent their first IVF/ICSI treatment cycle and initial ET were analyzed. Clinical pregnancy was defined as at least one gestational sac in the uterus identified by ultrasonography 35 days after ET. Miscarriage was defined as the loss of a clinical pregnancy before 28 weeks of gestation. Live birth was defined as the delivery of at least one living fetus after 28 weeks of gestation. Preterm delivery was defined as the delivery of a living fetus before 37 weeks of gestation.

Adverse pregnancy outcomes were analyzed among all deliveries after cumulative ET cycles. The incidence of pregnancy complications, including gestational diabetes mellitus and gestational hypertension, was evaluated. Low birth weight (LBW) was defined as birth weight < 2.5 kg. Large for gestational age (LGA) was defined as birth weight > 4.0 kg.

### Statistical analysis

Continuous variables were expressed as medians (interquartile range) when the data did not follow a Gaussian distribution, and as means (standard deviation) for normally distributed data. Categorical variables were presented as numbers (percentages). Student’s t-test and chi-square test were used to compare the differences in continuous and categorical variables, respectively, between the two groups. Comparisons between continuous variables without normal distributions were performed using the Mann-Whitney U test. Logistic regression analysis was conducted to calculate odds ratios (ORs) with 95% confidence intervals (CIs) for adjusting relevant factors. All statistical analyses were performed using SPSS version 24.0 software (Armonk, NY: IBM Corp.), and the results were considered statistically significant at values of *P* < 0.05.

## Results

The characteristics of the women analyzed are shown in Table 1. Significant differences in thyroid function were observed between the TC and control groups. The FT4 level was significantly higher (mean [SD]: 1.5 [0.2] vs. 1.2 [0.1], *P* < 0.001) and TSH level was significantly lower in the TC group than in the control group (median [interquartile range]: 0.5 [0.2–1.2] vs. 1.9 [1.4–2.6], *P* < 0.001). There were no differences in age, BMI, duration of infertility, basal hormone levels, and antral follicle count between the two groups (Table 1).

**Table 1:** Patients’ baseline characteristics

Characteristics	TC group (*n* = 64)	Control group^a^ (*n* = 320)	*P*-value
Age, mean (SD), years	33.8 (4.3)	33.6 (3.9)	0.774
Body mass index, mean (SD), kg/m^2^	23.1 (3.0)	23.0 (2.8)	0.688
Duration of infertility, median (IQR),y	3 (2–5)	3 (2–6)	0.531
Basal FSH, median (IQR), mIU/ml	7.1 (5.2–9.0)	6.6 (5.4–7.9)	0.237
Basal LH, median (IQR), mIU/ml	3.3 (2.4–4.2)	3.7 (2.6–5.2)	0.054
Basal estradiol, median (IQR), pmol/L	163 (126–221)	159 (128–203)	0.401
Antral follicle count in both ovaries, median (IQR)	11 (8–15)	10 (7–14)	0.398
FT4, mean (SD), ng/dl	1.5 (0.2)	1.2 (0.1)	< 0.001*
TSH, median (IQR), mIU/L	0.5 (0.2–1.2)	1.9 (1.4–2.6)	< 0.001*

*Abbreviations*: *TC* thyroid cancer, *IQR* interquartile range, *FSH* follicle-stimulating hormone, *LH* luteinizing hormone, *FT4* free thyroxine, *TSH* thyroid-stimulating hormone

^a^Patients in the control group were individually matched to a single case at a ratio of 5:1 for age, body mass index, concomitant infertility factors (polycystic ovarian syndrome, endometriosis, tubal factor, or male factor), years of IVF/ICSI treatment, protocols for controlled ovarian stimulation (ultralong-term, long-term, antagonist, short-term, or mini-stimulation protocol), and ET procedures (fresh ET or frozen ET)

*P < 0.05

With a similar dose of gonadotropin treatment, the hormone levels (LH, estradiol, progesterone) on the HCG trigger day were similar in the two groups; however, the number of retrieved oocytes was significantly lower in the TC group than in the control group (median [interquartile range]: 9  [6–14] vs. 12 [8–17], *P* = 0.025). The percentage of insemination cycles compared to the ICSI cycles was higher in the control group than in the TC group (56.3% vs. 69.7%, *P* = 0.036). The number of high-grade embryos was significantly lower in the TC group than in the control group (median [interquartile range]: 3 [2–5.8] vs. 4.5 [2–8], *P* = 0.046) (Table 2).

**Table 2:** In vitro fertilization and embryo transfer data between the TC and control groups

Characteristics	TC group (*N* = 64)	Control group (*N* = 320)	*P* value
Gonadotropin dose, median (IQR), IU	2175 (1500–2925)	2175 (1575–2850)	0.794
LH on hCG trigger day, median (IQR), mIU/ml	1.4 (0.8–3.1)	1.3 (0.7–2.8)	0.601
Estradiol on hCG trigger day, median (IQR), mIU/ml	5446 (3466–12,204)	6969.5 (4363.8–12,281.3)	0.121
Progesterone on hCG trigger day, median (IQR), pmol/L	2.1 (1.5–3.1)	2.3 (1.6–3.1)	0.458
No. of retrieved oocytes per cycle, median (IQR)	9 (6–14)	12 (8–17)	0.025*
Fertilization, no. (%)			
IVF	36 (56.3)	223 (69.7)	0.036*
ICSI	28 (43.8)	97 (30.3)	
High-quality embryos, median (IQR)^a^	3 (2–5.8)	4.5 (2–8)	0.046*
No. of embryos transferred, No. (%)			
1	15 (23.4)	52 (16.3)	0.212
2	48 (75.0)	267 (83.4)	
3	1 (1.6)	1 (0.3)	

*Abbreviations*: *IQR* interquartile range, *GnRH* gonadotropin-releasing hormone, *HCG* human chorionic gonadotropin, *ICSI* intracytoplasmic sperm injection, *IVF* in vitro fertilization

* *P* < 0.05

^a^ Embryos were evaluated on the third day after fertilization. Good-quality embryos all developed from two pronuclei zygotes and met the following criteria: (1) had > 5 blastomeres; (2) the size difference was < 20%; and (3) fragmentation was < 50%

Table 3 lists the pregnancy outcomes of the TC and control groups. There were no significant differences between the two groups in the rate of clinical pregnancy (35.9% vs. 34.7%, *P* = 0.848), miscarriage (4.3% vs. 10.8%, *P* = 0.466), live birth (34.4% vs. 30.9%, *P* = 0.589), or preterm delivery (9.1% vs. 10.1%, *P* > 0.999). The birth weight was also similar between the two groups. After adjusting for FT4, TSH, and methods for fertilization, no significant difference was observed between the TC and control groups in clinical pregnancy and live birth rates (OR, 1.49 and 1.43; 95% CI, 0.72–3.06 and 0.69–2.99; *P* = 0.286 and 0.337, respectively) (Table 4).

**Table 3:** Pregnancy outcomes after first IVF/ICSI treatment between the TC and control groups

Outcomes	TC group (*n* = 64)	Control group (*n* = 320)	*P*-value
Clinical pregnancy^a^, No. (%)	23/64 (35.9)	111/320 (34.7)	0.848
Miscarriage^b^, No. (%)	1/22 (4.3)	12/111 (10.8)	0.466^#^
Live birth^c^, No. (%)	22/64 (34.4)	99/320 (30.9)	0.589
Preterm delivery^d^, No. (%)	2/22 (9.1)	10/99 (10.1)	> 0.999^e^
Birth weight, g			
Singleton	3469.4 ± 372.3	3336.5 ± 543.4	0.327
Twin	2486.3 ± 657.2	2633 ± 384	0.390

*Abbreviations*: *TC* thyroid cancer, *ICSI* intracytoplasmic sperm injection, *IVF* in vitro fertilization

^a^Clinical pregnancy was defined as at least one gestational sac in the uterus at 35 days after embryo transfer as identified by ultrasonography

^b^Miscarriage was defined as loss of clinical pregnancy before 28 weeks’ gestation

^c^Live birth was defined as delivery of at least one living fetus beyond 28 weeks of gestation

^d^Preterm delivery was defined as delivery of living fetus before 37 weeks

^e^Fisher’s exact test

**Table 4:** Multivariate logistic regression analysis of factors associated with pregnancy outcomes after first IVF/ICSI treatment

Factors	Clinical pregnancy		Live birth	
	OR (95% CI)	*P*-Value	OR (95% CI)	*P*-Value
Group				
Control (reference)	N/A	N/A	N/A	N/A
TC	1.49 (0.72–3.06)	0.286	1.43 (0.69–2.99)	0.337
FT4	0.38 (0.11–1.40)	0.146	0.58 (0.16–2.14)	0.413
TSH	1.05 (0.82–1.36)	0.691	1.05 (0.81–1.36)	0.720
Fertilization				
IVF (reference)	N/A	N/A	N/A	N/A
ICSI	0.86 (0.55–1.36)	0.518	1.02 (0.64–1.62)	0.944

*Abbreviations*: *FT4* free thyroxine, *TSH* thyroid-stimulating hormone, *OR* odds ratio, *CI* confidence interval, *TC* thyroid cancer

After the first IVF/ICSI treatment cycle, 22 women in the TC group and 99 women in the control group had successful deliveries. Other women without delivery were followed up after subsequent IVF/ICSI or frozen ET cycles. A total of 35 women in the TC group and 168 women in the control group had successful deliveries. There was no significant difference in the occurrence of adverse obstetrics outcomes, including multiple gestation, preterm delivery, gestational diabetes mellitus, gestational hypertension, LBW, and LGA, between the TC and control groups (Table 5).

**Table 5:** Adverse pregnancy outcomes among all deliveries after cumulative transfer cycles

	TC group (*N* = 35)	Control group (*N* = 168)	*P* value
Multiple gestation, no. (%)	6/35 (17.1)	24/168 (14.3)	0.665
Preterm delivery, no. (%)	2/35 (5.7)	14/168 (8.3)	> 0.999
Gestational diabetes mellitus, no. (%)	2/35 (5.7)	14/168 (8.3)	> 0.999
Gestational hypertension, no. (%)	1/35 (2.9)	5/168 (3.0)	> 0.999
No. of infants	41	192	
Neonatal sex-male, no. (%)	21/41 (51.2)	109/192 (56.8)	0.516
LBW, no. (%)	7/41 (17.1)	21/192 (10.9)	0.291
LGA, no. (%)	3/41 (7.3)	13/192 (6.8)	> 0.999

*LBW* low birth weight, *LGA* large for gestational age, *TC* thyroid cancer

## Discussion

In this study, a significant difference was observed in the number of retrieved oocytes and high-grade embryos between the TC and control groups, suggesting that a history of TC may affect follicle growth and development during IVF/ICSI. The mechanism through which a history of TC affects follicle development remains unclear, but abnormal levels of thyroid hormone during IVF/ICSI may be involved. Many studies have shown that thyroid hormone plays an important role in regulating reproductive function and that both hyperthyroidism and hypothyroidism are associated with menstrual disturbance and irregular ovulation [8]. Navarro et al. and Aghajanova et al. detected the expression of thyroid hormone and TSH receptors in human ovarian tissues [9, 10]. Several previous studies demonstrated that thyroid hormones regulated follicle development and enhanced FSH-induced follicle growth, which may be achieved by promoting granulosa cell proliferation, decreasing granulosa cell apoptosis and affecting steroidogenesis [11–14]. Insufficient thyroid hormone secretion affects follicular development; however, excess thyroid hormone may also adversely affect follicular growth by altering the functionality of other hormones. Previous studies have reported that the levels of FSH, LH, and estradiol were higher and the androgen metabolism was changed in women with hyperthyroidism [15]. In our study, all patients in the TC group underwent thyroidectomy and were subsequently treated with thyroid hormone replacement before COS. Compared to the control group, the FT4 level was significantly higher, and TSH level was significantly lower in the TC group before IVF/ICSI. Abnormal thyroid hormone and TSH levels may affect follicular growth and development during COS and lead to a decreased number of retrieved oocytes and high-grade embryos.

The thyroid hormone plays an important role in the process of implantation and pregnancy maintenance. Aghajanova et al. demonstrated the presence of thyroid hormone and TSH receptors in the human endometrium, and Kokabashi et al. found that thyroid hormone facilitates the decidualization of human endometrial cells by affecting the expression of many transcription factors crucial for decidualization [16, 17]. After implantation, increased production of thyroid hormone plays a crucial role in maintaining a healthy pregnancy. During the first half of the pregnancy, the fetal thyroid is not mature to produce sufficient thyroid hormone and fetal development highly depends on the transfer of maternal thyroid hormone [18]. TC treatment impairs the normal thyroid function, which may affect the implantation and pregnancy outcomes. Previous studies have investigated whether a history of TC treatment affects the chances of pregnancy and a meta-analysis conducted by Busnelli et al. failed to show that TC treatment had any influence [19]. However, studies focused on the effect of a history of TC on pregnancy outcomes after IVF/ICSI are lacking. In our study, no significant difference was observed in the pregnancy outcomes after the first IVF/ICSI, including the rates of clinical pregnancy, miscarriage, live birth, preterm delivery, and birth weight between the TC and control groups. After adjusting for FT4, TSH, and fertilization procedures, there was also no significant difference in the rates of clinical pregnancy and live birth between the two groups. Our findings provide evidence that a history of TC does not affect the pregnancy outcomes after IVF/ICSI.

A recent study reported a higher risk of preterm delivery and LBW in cancer survivors; however, the study did not perform a subgroup analysis stratified by cancer type [20]. We analyzed the adverse pregnancy outcomes among all deliveries between the TC and control groups. We did not find an increased risk of multiple gestation, preterm delivery, gestational diabetes mellitus, and gestational hypertension. There was also no increase in the prevalence of infants with LBW or infants who were LGA in women with a history of TC compared with the control group, in accordance with the findings of previous studies. Two previous studies evaluated the risk of adverse pregnancy outcomes in survivors with different types of cancers and demonstrated that, unlike gynecological cancer and Non-Hodgkin lymphoma, the risk of adverse pregnancy outcomes was not increased in women with a history of TC [21, 22]. A recent study based on populations with a history of TC also failed to find a difference in the occurrence of preterm delivery, LBW, and LGA between women with a history of TC and their TC-free counterparts [23]. Thus, a history of TC was shown not to be a risk factor for the adverse pregnancy outcomes after IVF/ICSI.

## Conclusions

Our study demonstrates that a history of TC does not affect the pregnancy outcomes and does not increase the risk of adverse obstetric outcomes, but it does decrease the number of retrieved oocytes and high-grade embryos in this patient group. Our study findings provide valuable evidence for clinical strategies in infertile patients with TC. However, due to the limited number of women with a history of TC undergoing IVF/ICSI, the conclusions of our study need to be further confirmed in a study with a larger sample size.

## Data Availability

The datasets used or analyzed during the current study are available from the corresponding author on reasonable request.

## Abbreviations

TC: Thyroid cancer
IVF/ICSI: in Vitro fertilization/intracytoplasmic sperm injection
COS: Controlled ovarian stimulation
IVF: In vitro fertilization
ET: Embryo transfer
GnRH: Gonadotropin-releasing hormone
HCG: Human chorionic gonadotropin
TSH: Thyroid-stimulating hormone
FT4: Free thyroxine
TPOAb: Thyroid peroxidase antibody
TGAb: Thyroglobulin antibody
FSH: Follicle-stimulating hormone
LH: Luteinizing hormone
LBW: Low birth weight
LGA: Large for gestational age
ORs: Odds ratios

## References

[1] Cabanillas ME, McFadden DG, Durante C (2016). Thyroid cancer. Lancet.

[2] Zamora-Ros R, Rinaldi S, Biessy C, Tjønneland A, Halkjaer J, Fournier A, Boutron-Ruault MC, Mesrine S, Tikk K, Fortner RT, Boeing H, Förster J, Trichopoulou A, Trichopoulos D, Papatesta EM, Masala G, Tagliabue G, Panico S, Tumino R, Polidoro S, Peeters PHM, Bueno-de-Mesquita HB, Weiderpass E, Lund E, Argüelles M, Agudo A, Molina-Montes E, Navarro C, Barricarte A, Larrañaga N, Manjer J, Almquist M, Sandström M, Hennings J, Tsilidis KK, Schmidt JA, Khaw KT, Wareham NJ, Romieu I, Byrnes G, Gunter MJ, Riboli E, Franceschi S (2015). Reproductive and menstrual factors and risk of differentiated thyroid carcinoma: the EPIC study. Int J Cancer.

[3] Ding DC, Chen W, Wang JH, Lin SZ, Sung FC (2019). Thyroid cancer risk in women with infertility and association with fertility medications in Taiwan. Cancer.

[4] Alexander EK, Pearce EN, Brent GA, Brown RS, Chen H, Dosiou C, Grobman WA, Laurberg P, Lazarus JH, Mandel SJ, Peeters RP, Sullivan S (2017). 2017 guidelines of the American Thyroid Association for the diagnosis and Management of Thyroid Disease during Pregnancy and the postpartum. Thyroid.

[5] Veiga E, Treviño M, RomayAB ND, Trastoy R, Macía M (2019). Prevalence of genital Mycoplasma and response to eradication treatment in patients undergoing assisted reproductive techniques. Rev Esp Quimioter.

[6] Ricci S, De Giorgi S, Lazzeri E, Luddi A, Rossi S, Piomboni P (2018). Impact of asymptomatic genital tract infections on in vitro fertilization (IVF) outcome. PLoS One.

[7] The Istanbul consensus workshop on embryo assessment: proceedings of an expert meeting. Hum Reprod. 2011;26(6):1270–83. 10.1093/humrep/der037. 10.1093/humrep/der037 21502182

[8] Krassas GE, Poppe K, Glinoer D (2010). Thyroid function and human reproductive health. Endocr Rev.

[9] Aghajanova L, Lindeberg M, Carlsson IB, Stavreus-Evers A, Zhang P, Scott JE, Hovatta O, Skjöldebrand-Sparre L (2009). Receptors for thyroid-stimulating hormone and thyroid hormones in human ovarian tissue. Reprod BioMed Online.

[10] López Navarro E, Ortega FJ, Francisco-Busquets E, Sabater-Masdeu M, Álvarez-Castaño E, Ricart W, Fernández-Real JM (2016). Thyroid hormone receptors are differentially expressed in granulosa and cervical cells of infertile women. Thyroid.

[11] Di Paolo V, Mangialardo C, Zacà C, Barberi M, Sereni E, Borini A (2020). Thyroid hormones T3 and T4 regulate human luteinized granulosa cells, counteracting apoptosis and promoting cell survival. J Endocrinol Investig.

[12] Canipari R, Mangialardo C, Di Paolo V, Alfei F, Ucci S, Russi V, Santaguida MG (2019). Thyroid hormones act as mitogenic and pro survival factors in rat ovarian follicles. J Endocrinol Investig.

[13] Liu J, Tian Y, Ding Y, Heng D, Xu K, Liu W, Zhang C (2017). Role of CYP51 in the regulation of T3 and FSH-induced steroidogenesis in female mice. Endocrinology.

[14] Zhang C, Guo L, Zhu B, Feng Y, Yu S, An N, Wang X (2013). Effects of 3, 5, 3′-triiodothyronine (t3) and follicle stimulating hormone on apoptosis and proliferation of rat ovarian granulosa cells. Chin J Physiol.

[15] Krassas GE, Markou KB (2019). The impact of thyroid diseases starting from birth on reproductive function. Hormones.

[16] Aghajanova L, Stavreus-Evers A, Lindeberg M, Landgren BM, Sparre LS, Hovatta O (2011). Thyroid-stimulating hormone receptor and thyroid hormone receptors are involved in human endometrial physiology. Fertil Steril.

[17] Kakita-Kobayashi M, Murata H, Nishigaki A, Hashimoto Y, Komiya S, Tsubokura H (2020). Thyroid hormone facilitates in vitro decidualization of human endometrial stromal cells via thyroid hormone receptors. Endocrinology.

[18] Korevaar TIM, Medici M, Visser TJ, Peeters RB (2017). Thyroid disease in pregnancy: new insights in diagnosis and clinical management. Nat Rev Endocrinol.

[19] Busnelli A, Vitagliano A, Mensi L, Acerboni S, Bulfoni A, Filippi F, Somigliana E (2020). Fertility in female cancer survivors: a systematic review and meta-analysis. Reprod BioMed Online.

[20] Kao WH, Kuo CF, Chiou MJ, Liu YC, Wang CC, Hong JH, Hsu JT, Chiang YJ, Chuang YF (2020). Adverse birth outcomes in adolescent and young adult female cancer survivors: a nationwide population-based study. Br J Cancer.

[21] Anderson C, Engel SM, Mersereau JE, Black KZ, Wood WA, Anders CK (2017). Birth outcomes among adolescent and young adult cancer survivors. JAMA Oncol.

[22] Hartnett KP, Ward KC, Kramer MR, Lash TL, Mertens AC, Spencer JB, Fothergill A, Howards PP (2017). The risk of preterm birth and growth restriction in pregnancy after cancer. Int J Cancer.

[23] Cho GJ, Kim SY, Lee HC, Lee KM, Han SW, Oh MJ, Woodruff TK (2019). Risk of adverse obstetric outcomes and the abnormal growth of offspring in women with a history of thyroid cancer. Thyroid.

